# Supplementary material for: Phthalates exposure and serum uric acid level in patients with Crohn’s disease: A cross-sectional study
Source: PLoS One. 2026 Mar 3;21(3):e0343097. doi: 10.1371/journal.pone.0343097 (PMC12956089; doi:10.1371/journal.pone.0343097)
Supplement: S1 Table — (DOCX) [file pone.0343097.s001.docx]

**Table S1. Settings for Multiple Reaction Monitoring (MRM) Mode.**

| **Metabolites** | **Retention time**  **(min)** | **Precursor ion** | **Product ion** | **Collision**  **Energy (V)** |
| --- | --- | --- | --- | --- |
| MMP | 16.76 | 237 | 89 | 15 |
| MEP | 17.87 | 251 | 75 | 15 |
| MiBP | 19.79 | 223 | 75 | 15 |
| MnBP | 20.43 | 223 | 75 | 15 |
| MEHP | 24.86 | 221 | 73 | 15 |
| MBzP | 26.85 | 179 | 105 | 20 |
| MOP | 26.87 | 223 | 75 | 15 |
| MEOHP | 28.51 | 221 | 73 | 15 |
| MEHHP | 29.36 | 221 | 73 | 15 |
| MECPP | 31.40 | 221 | 73 | 15 |
| MEHP-C4 | 24.84 | 225 | 73 | 15 |
| MEHHP-C4 | 29.35 | 225 | 73 | 15 |

MMP: monomethyl phthalate; MEP: monoethyl phthalate; MiBP: monoisobutyl phthalate; MEHP: mono-(2-ethylhexyl) phthalate; MnBP: mono-n-butyl phthalate; MBzP: monobenzyl phthalate; MOP:mono-n-octyl phthalate; MEOHP: mono-(2-ethyl-5-oxohexyl) phthalate; MEHHP: mono-(2-ethyl-5-hydroxyhexyl) phthalate; MECPP: mono-(2-ethyl-5-carboxypentyl) phthalate.
