# Supplementary material for: Phthalates exposure and serum uric acid level in patients with Crohn’s disease: A cross-sectional study
Source: PLoS One. 2026 Mar 3;21(3):e0343097. doi: 10.1371/journal.pone.0343097 (PMC12956089; doi:10.1371/journal.pone.0343097)
Supplement: S2 Table — (DOCX) [file pone.0343097.s002.docx]

## **Table S2. The Quality Control of Urinary PAE Metabolites Qualification.**

| **Urinary PAE metabolites** | **LOD (ng/mL)** | **Spike recovery (%)** | | | | | **RSD (%)** | **Intra-assay CV (%)** | **Inter-assay CV (%)** |
| --- | --- | --- | --- | --- | --- | --- | --- | --- | --- |
|  |  | **Low (5ng/ml)** | **Median (10ng/ml)** | **High (50ng/ml)** | **Average** | |  |  |  |
| MMP | 1.0 | 93.2 | 89.8 | 85.7 | | 89.6 | 3.9 | 6.1 | 12.5 |
| MEP | 0.4 | 86.5 | 94.1 | 88.0 | | 89.5 | 2.9 | 6.3 | 8.2 |
| MiBP | 0.2 | 74.8 | 81.7 | 107.1 | | 84.0 | 1.6 | 4.0 | 7.0 |
| MBP | 0.2 | 72.2 | 83.4 | 96.5 | | 87.9 | 2.7 | 4.2 | 8.0 |
| MBzP | 0.2 | 71.9 | 70.0 | 73.0 | | 71.6 | 3.5 | 2.5 | 4.5 |
| MOP | 0.2 | 78.0 | 81.1 | 85.3 | | 81.5 | 6.0 | 4.9 | 6.3 |
| MEHP | 0.4 | 89.3 | 90.6 | 94.1 | | 91.3 | 0.7 | 3.4 | 5.0 |
| MEOHP | 1.0 | 73.8 | 73.8 | 81.4 | | 76.3 | 4.1 | 4.3 | 7.3 |
| MEHHP | 0.2 | 78.4 | 95.0 | 93.2 | | 88.9 | 0.6 | 3.2 | 7.4 |
| MECPP | 2.0 | 57.1 | 64.7 | 61.1 | | 61.0 | 9.0 | 9.5 | 14.9 |

Abbreviation: LOD, limit of detection; RSD, relative standard deviation; CV, Coefficient of variation.
