# Supplementary material for: Phthalates exposure and serum uric acid level in patients with Crohn’s disease: A cross-sectional study
Source: PLoS One. 2026 Mar 3;21(3):e0343097. doi: 10.1371/journal.pone.0343097 (PMC12956089; doi:10.1371/journal.pone.0343097)
Supplement: S3 Table — (DOCX) [file pone.0343097.s003.docx]

**Table S3. Stepwise Multivariate Linear Regression for the Interaction of mPAEs and HBI for SUA.**

| **mPAEs** | **Male (N=88)** | **Female (N=29)** |
| --- | --- | --- |
|  | **SUA level**  **(ln SUA)** | **SUA level**  **(ln SUA)** |
| **MMP*HBI** | / | / |
| **MEP*HBI** | / | / |
| **MIBP*HBI** | / | / |
| **MBP*HBI** | / | / |
| **MEHP*HBI** | / | / |
| **MBzP*HBI** | / | / |
| **MOP*HBI** | / | / |
| **MEOHP*HBI** | / | / |
| **MEHHP*HBI** | / | / |
| **MECPP*HBI** | / | / |
| **Total mPAEs*HBI** | / | / |
| **LMW mPAEs*HBI** | / | / |
| **HMW mPAEs*HBI** | / | / |
| **mDEHP*HBI** | / | / |

None of the interaction terms were included in the linear model.
