# Supplementary material for: Phthalates exposure and serum uric acid level in patients with Crohn’s disease: A cross-sectional study
Source: PLoS One. 2026 Mar 3;21(3):e0343097. doi: 10.1371/journal.pone.0343097 (PMC12956089; doi:10.1371/journal.pone.0343097)
Supplement: S4 Table — (DOCX) [file pone.0343097.s004.docx]

## Table S4. The Overall Associations of the Mixture of Ten Phthalate Metabolites with SUA Level and Hyperuricemia in Male CD Patients.

| **Quantile of mixture** | **Dependent variable (In SUA)** | | **Dependent variable (Hyperuricemia)** | |
| --- | --- | --- | --- | --- |
|  | **Estimated** | **Standard Deviation** | **Estimated** | **Standard Deviation** |
| 25% | -0.107 | 0.020 | -0.495 | 0.212 |
| 30% | -0.086 | 0.016 | -0.406 | 0.162 |
| 35% | -0.069 | 0.014 | -0.331 | 0.132 |
| 40% | -0.050 | 0.010 | -0.246 | 0.092 |
| 45% | -0.021 | 0.006 | -0.110 | 0.048 |
| 50% | 0.000 | 0.000 | 0.000 | 0.000 |
| 55% | 0.025 | 0.006 | 0.090 | 0.059 |
| 60% | 0.064 | 0.014 | 0.242 | 0.124 |
| 65% | 0.095 | 0.002 | 0.445 | 0.212 |
| 70% | 0.148 | 0.029 | 0.575 | 0.281 |
| 75% | 0.201 | 0.037 | 0.753 | 0.389 |

Estimated changes were assessed by Bayesian kernel machine regression (BKMR) models when the mixture was fixed at a particular percentile ranging from 25th to 75th percentile compared to that at the 50th percentile. Models were adjusted for age, BMI and HBI.

Because the natural logarithm has transformed the SUA levels before being included in the BKMR analysis, the result (the level of change in SUA) is equal to e^(0.201+0.107) = 1.36. The another result (OR of hyperuricemia) is equal to (0.753+0.495) = 1.25.
