# Supplementary material for: Phthalates exposure and serum uric acid level in patients with Crohn’s disease: A cross-sectional study
Source: PLoS One. 2026 Mar 3;21(3):e0343097. doi: 10.1371/journal.pone.0343097 (PMC12956089; doi:10.1371/journal.pone.0343097)
Supplement: S5 Table — (DOCX) [file pone.0343097.s005.docx]

**Table S5. The Posterior Inclusion Probabilities in the Bayesian Kernel Machine Regression Model in Male CD Patients.**

| **mPAEs** | **PIP for ln SUA** | **PIP for hyperuricemia** |
| --- | --- | --- |
|  |  |  |
| MMP | 0.08464 | 0.53644 |
| MEP | 0.00236 | 0.55380 |
| MiBP | 0.00136 | 0.54680 |
| MBP | 0.09060 | 0.56256 |
| MBzP | 0.00560 | 0.47992 |
| MOP | 0.12876 | 0.52440 |
| MEHP | 0.02240 | 0.55120 |
| MEOHP | 0.48340 | 0.85856 |
| MEHHP | 0.28516 | 0.71740 |
| MECPP | 0.00248 | 0.47576 |
