# Supplementary material for: Phthalates exposure and serum uric acid level in patients with Crohn’s disease: A cross-sectional study
Source: PLoS One. 2026 Mar 3;21(3):e0343097. doi: 10.1371/journal.pone.0343097 (PMC12956089; doi:10.1371/journal.pone.0343097)
Supplement: S6 Table — (DOCX) [file pone.0343097.s006.docx]

**Table S6. Mediating Effects of 8-OHdG on the Association of mPAEs and SUA Level in Male CD Patients.**

| **mPAEs 8-OHdG** | **ACME** | **ADE** | **Total effect** | **Proportion Mediated (%)** |
| --- | --- | --- | --- | --- |
| **MMP** | 0.035 (0.003, 0.104)* | 0.220 (0.100, 0.409)* | 0.255 (0.136, 0.447)* | 13.84 |
| **MEP** | 0.006 (-0.001, 0.023) | 0.008 (-0.005, 0.059) | 0.014 (0.004, 0.068)* | 40.77 |
| **MIBP** | 0.009 (0.002, 0.028)* | 0.016 (-0.010, 0.056) | 0.026 (0.004, 0.074)* | 36.84 |
| **MBP** | 0.002 (-0.000, 0.005) | 0.009 (0.003, 0.017)* | 0.011 (0.005, 0.018)* | 14.69 |
| **MEHP** | 0.016 (-0.002, 0.066) | 0.021 (-0.030, 0.118) | 0.037 (-0.008, 0.140) | 43.45 |
| **MBzP** | 0.190 (-0.451, 0.865) | 0.845 (0.112, 1.779)* | 1.034 (0.450, 1.833)* | 18.33 |
| **MOP** | 0.362 (0.072, 0.883)* | 1.175 (0.082, 2.709)* | 1.537 (0.544, 3.058)* | 23.56 |
| **MEOHP** | 0.012 (-0.018, 0.054) | 0.069 (0.019, 0.221)* | 0.082 (0.045, 0.220)* | 15.12 |
| **MEHHP** | 0.007 (-0.002, 0.025) | 0.039 (0.013, 0.100)* | 0.046 (0.026, 0.104)* | 15.48 |
| **MECPP** | 0.008 (0.000, 0.024)* | 0.008 (-0.014, 0.054) | 0.015 (-0.003, 0.064) | 49.48 |
| **Total mPAEs** | 0.001 (-0.002, 0.004) | 0.008 (0.003, 0.016)* | 0.009 (0.005, 0.016)* | 5.56 |
| **mDEHP** | 0.001 (-0.000, 0.004) | 0.007 (0.002, 0.014)* | 0.008 (0.004, 0.015)* | 15.37 |
| **LMW mPAEs** | 0.003 (-0.002, 0.012) | 0.013 (0.001, 0.035)* | 0.016 (0.008, 0.036)* | 19.16 |
| **HMW mPAEs** | 0.003 (-0.002, 0.012) | 0.013 (0.001, 0.034)* | 0.016 (0.008, 0.036)* | 19.61 |

Data are median (95%CI). The mediation model was adjusted for age, BMI, and HBI; Estimated r was shown; Abbreviation: ACME, average causal mediation effect; ADE, average direct effect. * P-value < 0.05.
