# Supplementary material for: Phthalates exposure and serum uric acid level in patients with Crohn’s disease: A cross-sectional study
Source: PLoS One. 2026 Mar 3;21(3):e0343097. doi: 10.1371/journal.pone.0343097 (PMC12956089; doi:10.1371/journal.pone.0343097)
Supplement: S7 Table — (DOCX) [file pone.0343097.s007.docx]

**Table S7. Mediating Effects of 8-iso-PGF2α on the Association of mPAEs and SUA Level in Male CD Patients.**

| **mPAEs** | **ACME** | **ADE** | **Total effect** | **Proportion Mediated (%)** |
| --- | --- | --- | --- | --- |
| **MMP** | 0.045 (-0.003, 0.130) | 0.210 (0.077, 0.405)* | 0.255 (0.136, 0.447)* | 17.50 |
| **MEP** | 0.010 (0.001, 0.031)* | 0.004 (-0.011, 0.055) | 0.014 (0.004, 0.068)* | 70.08 |
| **MIBP** | 0.013 (0.000, 0.032)* | 0.012 (-0.010, 0.055) | 0.026 (0.004, 0.074)* | 51.56 |
| **MBP** | 0.002 (-0.000, 0.005) | 0.009 (0.002, 0.016)* | 0.011 (0.005, 0.018)* | 17.11 |
| **MEHP** | 0.015 (-0.003, 0.070) | 0.022 (-0.026, 0.103) | 0.037 (-0.008, 0.140) | 39.48 |
| **MBzP** | 0.279 (-0.667, 1.012) | 0.756 (0.197, 1.788)* | 1.034 (0.450, 1.833)* | 26.95 |
| **MOP** | 0.400 (0.016, 0.880)* | 1.137 (0.140, 2.658)* | 1.537 (0.544, 3.058)* | 26.05 |
| **MEOHP** | 0.015 (-0.028, 0.067) | 0.067 (0.014, 0.225)* | 0.082 (0.045, 0.220)* | 18.58 |
| **MEHHP** | 0.008 (-0.002, 0.029) | 0.037 (0.010, 0.101)* | 0.045 (0.026, 0.104)* | 17.73 |
| **MECPP** | 0.009 (-0.001, 0.026) | 0.006 (-0.015, 0.052) | 0.015 (-0.003, 0.064) | 58.60 |
| **Total mPAEs** | 0.000 (-0.002, 0.004) | 0.008 (0.003, 0.016)* | 0.009 (0.005, 0.016)* | 4.62 |
| **mDEHP** | 0.002 (-0.001,0.005) | 0.007 (0.002, 0.014)* | 0.008 (0.004, 0.015)* | 18.83 |
| **LMW mPAEs** | 0.004 (-0.003, 0.013) | 0.012 (0.001, 0.036)* | 0.016 (0.008, 0.036)* | 23.51 |
| **HMW mPAEs** | 0.004 (-0.003, 0.013) | 0.012 (0.001, 0.035)* | 0.016 (0.008, 0.036)* | 24.01 |

Data are median (95%CI). The mediation model was adjusted for age, BMI, and HBI; Estimated r was shown; Abbreviation: ACME, average causal mediation effect; ADE, average direct effect. * P-value < 0.05.
