# Supplementary material for: Phthalates exposure and serum uric acid level in patients with Crohn’s disease: A cross-sectional study
Source: PLoS One. 2026 Mar 3;21(3):e0343097. doi: 10.1371/journal.pone.0343097 (PMC12956089; doi:10.1371/journal.pone.0343097)
Supplement: S1 File — (DOCX) [file pone.0343097.s008.docx]

Measurement of phthalates

**1. Determination of urinary mPAEs**

***1.1. Reagents and apparatus***

Standards of MMP, MEP, MiBP, MBP, MEHP, MBzP, and MOP were purchased from Accustandard (New Haven, CT, USA). MEOHP, MEHHP, and MECPP were purchased from Cambridge Isotope Laboratories (Andover, MA, USA). The isotope-labeled internal standard of 1-OHNAP-D7 was purchased from C/D/N isotopes (Pointe-Claire, Quebec, Canada), that of 1-OHPYR-D9 was obtained from Toronto Research Chemicals (North York, Ontario, CAN), and that of MEHP-C4. MEHHP-C4 was obtained from Cambridge Isotope Laboratories (Andover, MA, USA).

Sodium acetate, acetonitrile, n-Hexane, and E-coli β-glucuronidase (> 100,000 units/mL) were purchased from ANPEL Laboratory Technologies (Shanghai, CHN). Ether and magnesium sulfate heptahydrate were purchased from Sinopharm Chemical Reagent (Shanghai, CHN). Acetic acid was obtained from Sigma-Aldrich (Saint Louis, MO, USA). Derivatization reagent N,O-bis(trimethylsilyl)-trifluoro acetamide (BSTFA) with 1% of trimethylchlorosilane (TMCS) was purchased from Regis Technologies (Morton Grove, IL, USA).

The gas chromatograph (GC) used in the present study was an 8890 Series GC coupled to a 7010B triple quadrupole mass spectrometry system from Agilent Technologies (Palo Alto, CA, USA). A 7693A automatic liquid sampler (Agilent) and MussHunter Workstation (Version 10.0.368) were used.

***1.2. Standard preparation and calibration procedure***

For PAEs, a concentration of 10 mg/mL native stock solution were prepared for MMP, MEP, MiBP, MBP, MEHP, MBzP, MOP, and 2 mg/mL for MEOHP, MEHHP, and MECPP. The mixture of all ten standards at a final concentration of 500 ng/mL was used as the working standard solutions.

The mixed working internal standard solution containing MEHP-C4, and MEHHP-C4 were prepared at a final concentration of 5 μg/mL. MEHP-C4 was used as the internal standard for MMP, MEP, MiBP, MBP, MEHP, MBzP, and MOP, while MEHHP-C4 for MEOHP, MEHHP, and MECPP. All the native stock and working solutions were stored at -20℃ until use. The calibration standards ranging in concentration from 2.5 ng/mL to 500 ng/mL for the high-exposure group and 0.25 ng/mL to 50 ng/mL for the low-exposure group were prepared fresh daily in water for calibration curves. The quality control (QC) materials were also prepared fresh daily in water at concentrations of 50 and 5 ng/mL.

***1.3. Enzymatic hydrolysis***

In 3 mL of urine sample, 20 μL of the internal standard mixtures (5 μg/mL) was spiked. To adjust the pH for enzyme activation, 1 mL of 0.5 M of sodium acetate solution (pH = 0.5) was added. Then 20 μL of β-glucuronidase was added, and the mixture was hydrolyzed at 37°C for 12 hours.

***1.4. Liquid-liquid extraction and derivatization***

To increase the ionic strength of the sample solution, 4.0g of magnesium sulfate heptahydrate was added once enzymatic hydrolysis is completed. The samples were then oscillated on the high-speed microplate shaker for 10 min (2100 rpm). For extraction, 1.5 mL of extracting solvent was added to a sample and mechanically shaken for 3 min. The organic phase was separated by centrifuging at 3000 rpm for 10 min and transferred into another polypropylene centrifugal tube. These procedures were repeated three times. Total volumes of about 4.5 mL of the extracted solvents were then vaporized at room temperature. After vaporization, 100 μL of BSFTA/TMCS (99: 1, v/v) was spiked in the sample tube and incubated at 90℃ for 45 min for alkoxylation.

***1.5. GC-MS determination***

Chromatographic separation was performed on an HP-5MS capillary column (30m length, 0.25mm internal diameter, 0.25 μm film thickness, Agilent Technologies, Inc., Santa Clarita, California, USA). For the carrier gas, a helium flow at 1.2 mL/min was used. A helium flow at 4 mL/min was used as quenching gas and a nitrogen flow at 1.5 mL/min as collision gas. The oven temperature was programmed as follows: 60℃ for 3 min, which was increased to 150℃ at a rate of 10℃/min and held for 3 min, then increased to 210℃ at a rate of 10℃/min and held for 5 min, finally increased to 310℃ at a rate of 10℃/min and held constant for 4 min. The injector was operated in the splitless mode at 280℃ (1 μL injection volume). The detector was operated in the multiple reaction monitoring mode (MRM) at a temperature of 250℃.

The limits of detection (LODs) for mPAEs ranged from 0.20 to 2.00 ng/mL, and values below the LODs were substituted with LOD/√2. The spike recoveries for urinary mPAEs ranged from 61.0% to 91.3%.
